# Targeted Expression to Liver of an antimiR-33 Sponge as a Gene Therapy Strategy against Hypercholesterolemia: In Vitro Study

**DOI:** 10.3390/cimb45090445

**Published:** 2023-08-24

**Authors:** Mariela Montaño-Samaniego, Jorge Sánchez-Cedillo, Amellalli Lucas-González, Diana M. Bravo-Estupiñan, Ernesto Alarcón-Hernández, Sandra Rivera-Gutiérrez, José Abraham Balderas-López, Miguel Ibáñez-Hernández

**Affiliations:** 1 Laboratorio de Terapia Génica, Departamento de Bioquímica, Escuela Nacional de Ciencias Biológicas, Instituto Politécnico Nacional, Mexico City 11340, Mexico; mariela.mont3091@gmail.com (M.M.-S.); sanchez20890@gmail.com (J.S.-C.); iiaiizz0412@gmail.com (A.L.-G.); diana.marcela.bravo94@gmail.com (D.M.B.-E.); 2 Laboratorio de Técnicas Fototérmicas, Departamento de Ciencias Básicas, Unidad Politécnica Interdisciplinaria de Biotecnología, Instituto Politécnico Nacional, Mexico City 07340, Mexico; jbalderasl@ipn.mx; 3 Laboratorio de Quimiosensibilidad Tumoral, Facultad de Microbiología, Universidad de Costa Rica, San Jose 11501-2060, Costa Rica; 4 Laboratorio de Genética Molecular, Departamento de Bioquímica, Escuela Nacional de Ciencias Biológicas, Instituto Politécnico Nacional, Mexico City 11340, Mexico; ealarcon@ipn.mx; 5 Laboratorio de Microbiología Molecular, Departamento de Microbiología, Escuela Nacional de Ciencias Biológicas, Instituto Politécnico Nacional, Mexico City 11340, Mexico; srivera@ipn.mx

**Keywords:** atherosclerosis, gene therapy, antimiR-33 sponge, ABCA1 and ABCG1 transporters

## Abstract

Atherosclerosis is the leading cause of cardiovascular diseases in Mexico and worldwide. The membrane transporters ABCA1 and ABCG1 are involved in the reverse transport of cholesterol and stimulate the HDL synthesis in hepatocytes, therefore the deficiency of these transporters promotes the acceleration of atherosclerosis. MicroRNA-33 (miR-33) plays an important role in lipid metabolism and exerts a negative regulation on the transporters ABCA1 and ABCG1. It is known that by inhibiting the function of miR-33 with antisense RNA, HDL levels increase and atherogenic risk decreases. Therefore, in this work, a genetic construct, pPEPCK-antimiR-33-IRES2-EGFP, containing a specific antimiR-33 sponge with two binding sites for miR-33 governed under the PEPCK promoter was designed, constructed, and characterized, the identity of which was confirmed by enzymatic restriction, PCR, and sequencing. Hep G2 and Hek 293 FT cell lines, as well as a mouse hepatocyte primary cell culture were transfected with this plasmid construction showing expression specificity of the PEPCK promoter in hepatic cells. An analysis of the relative expression of miR-33 target messengers showed that the antimiR-33 sponge indirectly induces the expression of its target messengers (ABCA1 and ABCG1). This strategy could open new specific therapeutic options for hypercholesterolemia and atherosclerosis, by blocking the miR-33 specifically in hepatocytes.

## 1. Introduction

Cardiovascular diseases (CVD) are the leading cause of death in Mexico and worldwide, and atherosclerosis is the most important risk factor. The number of people who suffer with atherosclerosis is increasing [1,2]. Lipoprotein imbalance, specifically an increase in low-density lipoproteins (LDL) and a decrease in high-density lipoproteins (HDL), has been shown to trigger atheroma formation [3,4,5]. The ABCA1 and ABCG1 membrane transporters participate in cholesterol transport outside of any cell type and favor the increase in HDL synthesis in hepatocytes, therefore a deficiency of these transporters promotes accelerated atherosclerosis [6,7,8]. The genetic expression of these transporters and many other biological processes are regulated by microRNAs (miRNAs or miRs). Family miR-33 is very well characterized as a proatherogenic miRNA and by its participation in lipid metabolism, by inhibiting the expression of ABCA1 and ABCG1, which indirectly up-regulates cholesterol generation and down-regulates HDL production [9,10,11]. There are two members of this family called miR-33a and miR-33b [12]. It is known that the overexpression of miR-33 reduces fatty acid oxidation, while its inhibition increases this metabolic pathway [10,13,14]. In addition, it has been reported that the absence of ABCA1 expression causes the intracellular accumulation of cholesterol and facilitates the progression of atherosclerosis [15]. Studies have been carried out to inhibit the function of miR-33 by using antisense oligonucleotides (ASO); with this, the suppression of the effect of miRNA was achieved and, therefore, there was a higher expression of the ABCA1 transporter, an observed increase in HDL and a decrease in total plasma cholesterol levels, reversing the effects of atherosclerosis in the murine model used [8,16,17,18,19,20]. However, the lack of specificity of such inhibition, and thus the presence of adverse effects due to high doses and continuous administration required, does not make it a safe therapeutic strategy [21,22]. To overcome the problem of multiple administrations of antisense oligonucleotides, ‘miRNA sponges’ were developed as another way to inhibit miRNA activity in which plasmids are generally used, expressing a high copy number of the anti-miRNA transcript, and are less vulnerable to nucleases [23,24]. miRNA sponges contain multiple miRNA-binding sites, which act as competitive inhibitors for miRNA-binding sites, however, its expression alone remains tissue-unspecific.

On the other hand, gene therapy is a treatment strategy that uses “therapeutic” nucleic acids introduced into the target cells to express themselves and cause a beneficial biological effect [25] for the treatment or prevention of genetic diseases. In order to achieve gene therapy, the genetic material must overcome various cellular barriers to reach the cytoplasm and enter the nucleus for appropriate expression. In this sense, the use of cationic liposomes as genetic vehicles is justified by their simple preparation, because they interact electrostatically with the genetic material of interest, for their property to transfect a variety of cell lines and by a low immunogenicity, which could allow safe application in vivo [26,27]. Cationic liposomes are ideal for transferring a therapeutic gene but are difficult to direct to a specific target, hence tissue-specific promoters have been used to direct the expression only in target cells where the promoter is active [25]. The phosphoenolpyruvate carboxykinase enzyme (PEPCK) is an enzyme that catalyzes the irreversible conversion of oxaloacetate to phosphoenolpyruvate, an important initial and specific step of hepatic gluconeogenesis [28,29] so the PEPCK gene promoter is functional only in liver cells [30]. Hence, this work aimed to design, construct, and characterize a recombinant plasmid pPEPCK-antimiR-33-IRES2-EGFP, containing a specific antimiR-33 sponge with two binding sites for miR-33 governed under the PEPCK promoter, to direct the expression of the sponge exclusively to liver cells, just as was demonstrated by in vitro transfections. Furthermore, an analysis of the relative expression of miR-33 target messengers showed that the antimiR-33 sponge indirectly induces the expression of its target messengers: ABCA1 and ABCG1 specifically in liver cells.

## 2. Materials and Methods

### 2.1. miRNA Sponge Design

#### 2.1.1. antimiR-33 Sponge Design and Composition

The antimiR-33 sponge sequence was designed using the Kluiver method, specifically the oligonucleotide duplex approach [31], with two perfect binding sites for miR-33a; according to miRBase (5′-GUGCAUUGUAGUUGCAUUGCA-3′), each binding site is the antisense sequence of miR-33a. The 5′ and 3′ ends of the oligonucleotide duplex, corresponding to the antimiR-33 sponge, consist of overhangs that are compatible with the SacI and XmaI restriction enzymes, respectively.

#### 2.1.2. In Silico Analysis of Sponge Specificity and Functionality

To optimize the sequence of the antimiR-33 sponge, the online miRNAsong software was used to analyze it with standard settings [32]; this was in order to determine the binding specificity and efficiency of miR-33. For this in silico analysis, the designed antimiR-33 sponge sequence was uploaded in the program and validated, then the sense and antisense oligonucleotides containing the two miR-33a binding sites was synthetized by the Biotechnology Institute (IBT-UNAM, Cuernavaca, Mexico).

### 2.2. Genetic Vector Design

The genetic vector design was carried out using the VectorNTI Advance^TM^ (Invitrogen, Waltham, MA, USA), beginning with the pIRES2-EGFP eukaryotic expression vector (BD Clontech, Mountain View, CA, USA), with a size of 5.3 Kb. The in silico construction was performed by releasing the CMV promoter and a fragment of the MCS with AseI, BglII, SacI, and XmaI restriction enzymes and by ligating the inserts of interest (PEPCK promoter and antimiR-33 sponge).

### 2.3. Genetic Vector Construction

#### 2.3.1. Oligonucleotide Duplex Generation

From the sense and antisense oligonucleotides (corresponding to the antimiR-33 sponge) diluted in nuclease-free water (500 ng/µL), an alignment was performed to obtain the double-stranded DNA fragments by subjecting the oligonucleotide pair to 95 °C for 10 min and gradually cooling them to room temperature, then storing at 4 °C. The formation of the duplex was verified through 2% agarose gel electrophoresis, in TBE regulator 1× (Invitrogen) at 90 V for 60 min, together with a 50 bp ladder. The gel was stained with ethidium bromide (0.5 µg/mL), then observed and imaged on the Kodak Gel Logic 100 Digital Imaging System Transilluminator (Kodak, Rochester, NY, USA).

#### 2.3.2. Primer Design and PEPCK Promoter Amplification

The primer design from which to amplify the PEPCK promoter fragment was performed using Vector NTI Advance^TM^ (Invitrogen), which contains AseI and BglII restriction enzyme cleavage sites: forward: 5′-CGCATTAATGCTTACAATCACCCCTCCC-3′ and reverse: 5′-AATAGATCTCAGAGCGTCTCGCC-3′ to give directionality to cloning and obtain the correct transcription for the therapeutic gene. Then, the PEPCK promoter was amplified by PCR from the pPEPCK-hGH vector (donation by Campos-Naciff, Begoña) using the PCR Master Mix (Promega, Madison, WI, USA).

#### 2.3.3. Preparation of Cloning Vector and Ligation Reaction

The pIRES2-EGFP vector and the amplified PEPCK promoter were digested by AseI and BglII, the digestion products were separated with 1% agarose gel electrophoresis, and the interest fragments were retrieved and purified using the QIAEX^TM^ II Gel Extraction Kit (QIAGEN, Hilden, Germany). Then, the open vector was ligated with the PEPCK promoter by a T4 DNA Ligase (Thermo Fisher Scientific, Waltham, MA, USA). Ligation reactions were performed in 20 µL with 1 µL 5 U/µL T4 ligase in the buffer supplied by the manufacturer. The recombinant pPEPCK-IRES2-EGFP was transformed in a vector/insert ratio of 1:5 by the heat shock method into *E. coli* DH5α competent cells and was isolated with the UltraClean^®^ Maxi Plasmid Prep Kit (MO BIO Labs, Carlsbad, CA, USA). Subsequently, the purified recombinant plasmid was digested by SacI and XmaI and then ligated with the oligonucleotide duplex of the antimiR-33 sponge by a T4 Ligase. This recombinant vector pPEPCK-antimiR-33-IRES2-EGFP was transformed in a vector/insert ratio of 1:20 into *E. coli* InvαF’ competent cells for amplification and was isolated by alkaline lysis with the UltraClean^®^ Maxi Plasmid Prep Kit (MO BIO Labs) and stored at 4 °C.

#### 2.3.4. Genetic Vector Characterization

The pPEPCK-antimiR-33-IRES2-EGFP DNA concentration and purity were validated with the Nanodrop 1000^TM^ Spectrophotometer (Thermo Fisher Scientific). The construction identity was confirmed by enzymatic restriction (with AseI, BglII, SacI, and XmaI) and PCR, then, agarose gel electrophoresis was used to verify the fragments’ size. In addition, this plasmid was submitted for Sanger automatic sequencing ABI Prism (IBT-UNAM) to confirm the insert sequence integrity. The obtained sequence was used to perform a local alignment analysis with the online software EMBOSS Water (https://www.ebi.ac.uk/Tools/psa/emboss_water/ accessed on 30 January 2020), comparing it with the designed recombinant plasmid sequence.

### 2.4. Evaluation of Tissue Specificity from antimiR-33 Sponge Expression

#### 2.4.1. Cell Cultures

To validate the effectiveness of the sponge transcript to bind to the desired miRNA, first Hek-293 FT (human embryonic kidney cells) and Hep G2 (human hepatocarcinoma cells) were cultivated in Dulbecco’s modified Eagle’s medium (DMEM, Sigma–Aldrich, St. Louis, MO, USA) with 10% fetal bovine serum (FBS, Sigma–Aldrich) at 37 °C in a 5% CO_2_-containing atmosphere. For transfection, Hek-293 FT and Hep G2 cells were passaged and plated at 50,000 cells per well in a 24-well plate (for expression specificity assay) and 100,000 cells per well in a 12-well plate (for mRNA assay) and incubated for 24 h at 37 °C in a 5% CO_2_-containing atmosphere, at 80% confluence, to be used in transfection with the recombinant pPEPCK-antimiR-33-IRES2-EGFP and the control pIRES2-EGFP plasmids.

#### 2.4.2. Hepatocyte Primary Cell Culture

The mouse hepatocytes culture was carried out by two cellular disintegration methods: mechanical and enzymatic disintegration, based on Freshney protocols [33], using Hepatocyte Wash Medium (Gibco™, Abingdon, UK) and following the considerations of the Official Mexican Standards NOM-062-ZOO-1999 [34] and NOM-033-ZOO-1995 [35]. Finally, the cell button was resuspended in 1 mL of Hepatocyte Medium (Sigma^TM^, Tokyo, Japan). Subsequently, 250,000 viable cells were seeded per well in a 6-well plate and incubated for 24 h at 37 °C in 5% CO_2_-containing atmosphere, at 80% confluence, to be used in transfection with the recombinant and the control plasmid.

#### 2.4.3. Cell Transfection

Transfection was carried out according to the instructions included with the Lipofectamine^TM^ 2000 (Thermo Fisher Scientific). Lipoplexes were obtained by mixing pDNA (pIRES2-EGFP or pPEPCK-antimiR-33-IRES2-EGFP) with Lipofectamine in no serum DMEM for plasmid quantities between 860 ng and 3.4 µg, according to the number-well plate.

Cells were divided into three groups: Cell lines (negative control), Lipofectamine^TM^ 2000 pIRES2-EGFP (positive control), and Lipofectamine^TM^ 2000 pPEPCK-antimiR-33-IRES2-EGFP (recombinant) and were transfected in two independent experiments by pouring culture medium from wells until the cells were barely covered and adding the lipoplex solution on each well, incubating for 2 h at the same culture conditions. After that, 500 μL, 1 and 2 mL of complete DMEM were added for incubation (for 24-well, 12-well, and 6-well plates, respectively). After a period of 72 h after transfection, reporter gene expression was directly evaluated using fluorescence microscopy in Axio Vert.A1 (Carl Zeiss, Oberkochen, Germany) with a 20× objective, and using the reporter gene EGFP, transfection efficiency was evaluated by comparing cells EGFP positive against total cells per field. Software Image J version 1.53t (Open Source Software (OSS) license) was used to count cells. Then, the transfected cells were cultured in G418 (Gibco) selection medium to screen for resistant cells since the pIRES2-EGFP backbone contains the Neomycin resistance gene. After 7 days, the positive cell clones were collected and used for subsequent mRNA assays.

#### 2.4.4. Total RNA Extraction

Total RNA was isolated from the negative control group cells and the transfected recombinant group cells, using TRIzol reagent^TM^ (Invitrogen), according to the manufacturer’s protocol. The RNA concentration and purity were determined using the Nanodrop 1000 spectrophotometer (Thermo Fisher Scientific) and the structural integrity was determined by denaturing electrophoresis in 1% agarose gel added with 1% sodium hypochlorite (Clorox^®^, Oakland, CA, USA) [36]. Three primers pairs were designed based on the sequence of the ABCA1, ABCG1, and GAPDH genes (access numbers: NM_005502.4, NM_016818.2, and NM_002046.7, respectively) to perform the qPCR: ABCA1 forward: 5′-AGACGCAAACACAAAAGTGGA-3′ and reverse: 5′-GCAGCAGCTGACATGTTTGT-3′; ABCG1 forward: 5′-GTTCTTCGATGAGCCCACCA-3′ and reverse: 5′-AGGACGTAAAGCTGGTCGAA-3′; and GAPDH forward: 5′-GGAAGGTGAAGGTCGGAGTC-3′ and reverse: 5′-ACATGTAAACCATGTAGTTGAGGTC-3′.

#### 2.4.5. RT-qPCR

RT reactions were performed using the Cloned AMV First-Strand cDNA Synthesis Kit (Invitrogen) and qPCR was performed using the QuantiNova SYBR Green PCR Kit (QIAGEN) on the Rotor-Gene Q series (QIAGEN), according to the manufacturer’s protocols. Thermocycling conditions were as follows: Initial denaturation at 95 °C for 2 min, followed by 35 cycles of 95 °C for 10 s and 60 °C for 20 s. Each reaction was independently tested two times. GAPDH was used as the internal control and the target genes levels of miR-33a were quantified using the comparative CT method (2^−ΔΔCt^) [37]. Equation (1) was used:

Gene expression = 2^−∆∆CT^ (1)

where:

∆∆C_T_ = (C_T,gene_ − C_T,GAPDH_)_treated_ − (C_T,gene_ − C_T,GAPDH_)_control_

#### 2.4.6. Statistical Analysis

Data are shown as the mean of *n* = 4 with respective SD. Statistical analysis was conducted using GraphPad Prism version 9.5.1 (GraphPad Software LLC, San Diego, CA, USA). The Shapiro–Wilk test was performed to determine the distribution type of the samples. A Brown–Forsythe and Welch ANOVA test followed by Dunnett’s post hoc test was used to evaluate the differences between the three groups, for those with normal distribution; on the other hand, the samples with non-normal distribution were analyzed with the non-parametric Kruskal–Wallis test followed by Dunn’s post-hoc tests. *p* ≤ 0.05 was considered to indicate a statistically significant difference.

## 3. Results

### 3.1. miRNA Sponge Design and In Silico Analysis

#### 3.1.1. antimiR-33 Sponge Design and Composition

The designed antimiR-33 sponge sequence contains two perfect binding sites for miR-33a, separated by a short 4 nt spacer sequence. The restriction enzymes sites at 5′ and 3′ ends of the oligonucleotide duplex work to give directionality to cloning (Table 1).

#### 3.1.2. In Silico Analysis of Sponge Specificity and Functionality

**Figure 1:**
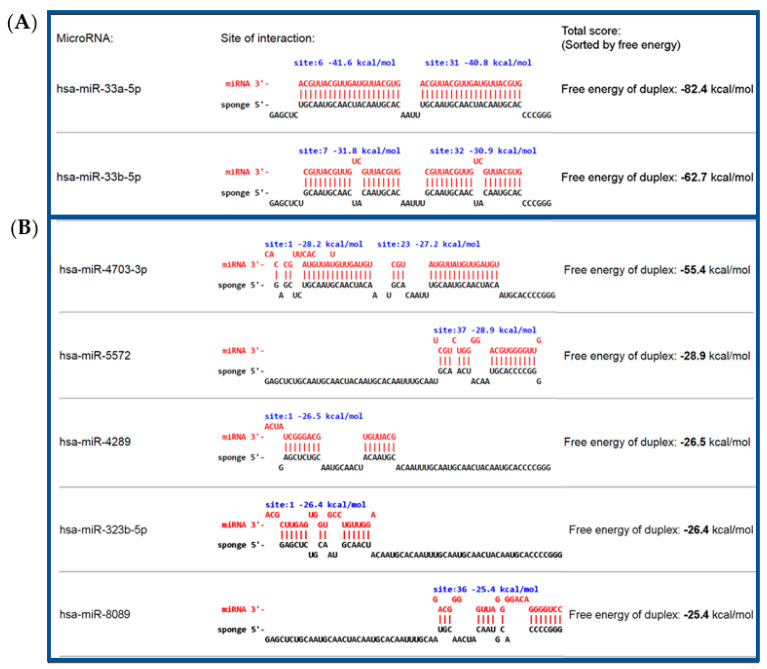
Functionality and specificity analysis of the antimiR-33 sponge sequence. (**A**) It is noted that miR-33a and b are joined more strongly to the antimiR-33 sponge; (**B**) other miRNAs can be joined to the sponge but in a partial way and with less affinity than with miR-33a and miR-33b. In addition, miR-4703 is only expressed in thyroid gland; miR-5572 is expressed in several human tissues, liver included; miR-4289 is not expressed in liver; miR-323b is expressed mainly in ovaries, pancreas and brain; and miR-8089 is expressed in several tissues, included in right lobe of liver [38].

The bioinformatic analysis for functionality of the antimiR-33 sponge sequence, showed two different ways of interacting with miR-33 in each binding site of the antimiR-33 sponge: a partial but hard interaction with member miR-33b, with a ΔG value of −62.7 kcal/mol, and another totally complete interaction with member miR-33a, with a ΔG value of −82.4 of kcal/mol (Figure 1A). In addition, it was found that it can be targeted by other miRNAs but mainly by miR-33a and miR-33b with more strength and specificity, in agreement with the obtained ∆G values; furthermore, most of those other miRNAs are not expressed in hepatocytes (Figure 1B).

### 3.2. Genetic Vector Design and Construction

The design and in silico construction of the recombinant vector pPEPCK-antimiR-33-IRES2-EGFP contains the antimiR-33 sponge and the green fluorescent protein gene, both sequences under the transcriptional control of the PEPCK promoter (Figure S1).

#### PEPCK Promoter and miRNA Sponge Cloning

The formation of the duplex (58 bp) was verified through gel electrophoresis, together with the single-stranded oligonucleotides (not shown). The genetic construction pPEPCK-antimiR-33-IRES2-EGFP contains the antimir-33 sponge sequence as a therapeutic sequence and the green fluorescent protein (EGFP) reporter gene, governed under the transcriptional control of the PEPCK promoter. The identity of this genetic construction was verified by enzymatic restriction (Figure S2A), PCR (Figure S2B), and sequencing, compared to the sequence of the designed plasmid (Figure S2C,D). The PEPCK promoter sequence was compared with the Genebank database, in which a 100% identity with the reported sequence was obtained (Access number: 362282), showing that both the therapeutic gene (antimiR-33 sponge) and the tissue-specific promoter that governs its expression (PEPCK) are correctly inserted in the recombinant plasmid.

### 3.3. Liver Tissue Specificity of the Genetic Construction pPEPCK-antimiR33-IRES2-EGFP

**Figure 2:**
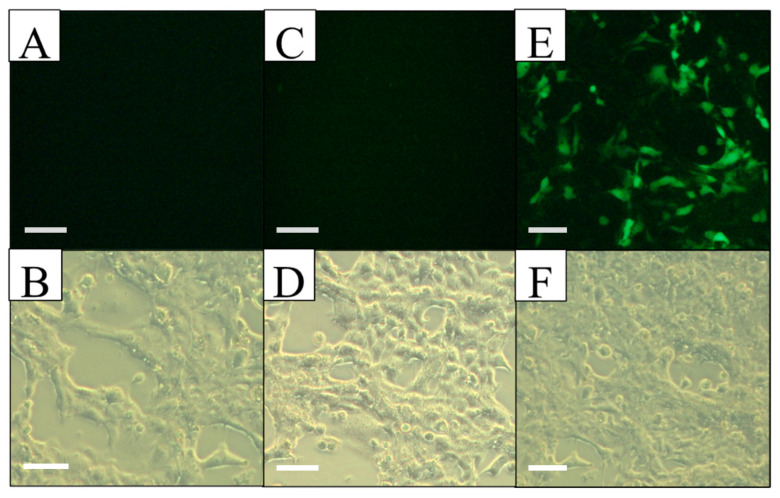
Cell morphology and EGFP expression specificity in Hek293 FT observed by light and fluorescent microscopy at 72 h post-transfection. (**A**,**B**) Non-transfected culture, observed with UV light (top) and in bright field (bottom), without EGFP expression. (**C**,**D**) Culture transfected with pPEPCK-antimiR-33-IRES2-EGFP, observed with UV light (top) and in bright field (bottom), EGFP expression is not detected. (**E**,**F**) Culture transfected with pIRES2-EGFP, observed with UV light (top) and in bright field (bottom), EGFP expression is observed. Scale bars = 50 µm.

**Figure 3:**
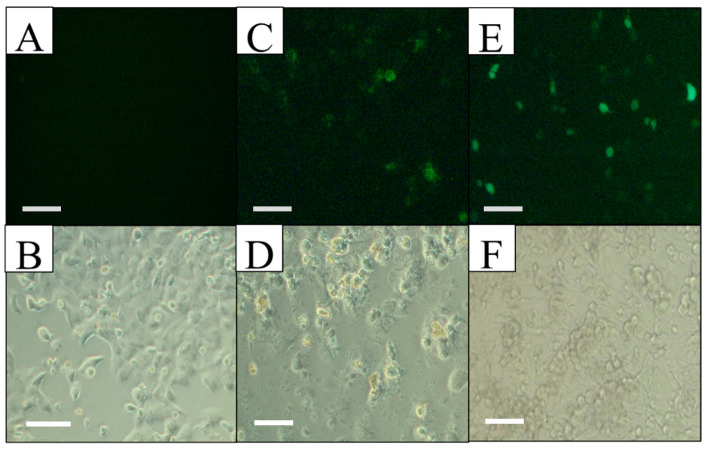
Cell morphology and EGFP expression specificity in Hep G2 observed by light and fluorescent microscopy at 72 h post-transfection. (**A**,**B**) Non-transfected culture, observed with UV light (top) and in bright field (bottom), without EGFP expression. (**C**,**D**) Culture transfected with pPEPCK-antimiR-33-IRES2-EGFP, observed with UV light (top) and in bright field (bottom), EGFP expression is observed. (**E**,**F**) Culture transfected with pIRES2-EGFP, observed with UV light (top) and in bright field (bottom), EGFP expression is observed. Scale bars = 50 µm.

**Figure 4:**
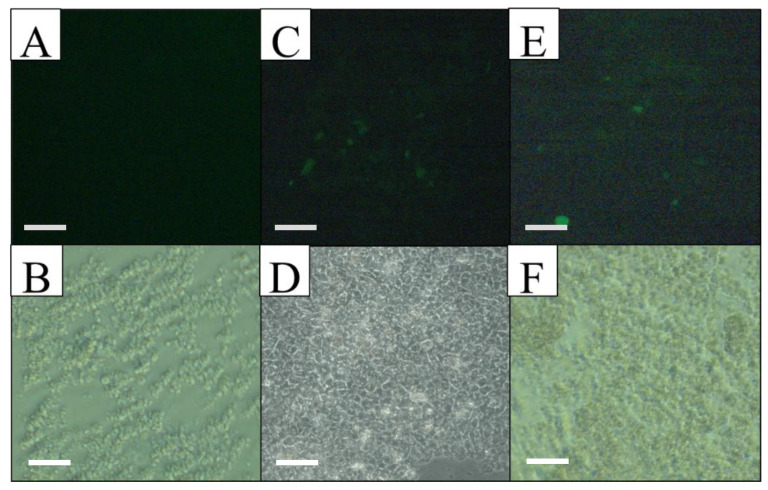
Cell morphology and EGFP expression specificity in the primary mouse hepatocyte culture, observed by light and fluorescent microscopy at 72 h post-transfection. (**A**,**B**) Non-transfected culture, observed with UV light (top) and in bright field (bottom), without EGFP expression. (**C**,**D**) Culture transfected with pPEPCK-antimiR-33-IRES2-EGFP, observed with UV light (top) and in bright field (bottom), EGFP expression is observed. (**E**,**F**) Culture transfected with pIRES2-EGFP, observed with UV light (top) and in bright field (bottom), EGFP expression is observed. Scale bars = 50 µm.

With this genetic vector, the Hep G2 and Hek 293 FT cell lines, as well as a primary mouse hepatocyte culture, were transfected. Reporter gene (EGFP) expression was directly evaluated 72 h after transfection, using fluorescence microscopy, taking advantage of the fluorescence activity of the pIRES2-EGFP plasmid. Since the EGFP gene is found downstream of the antimiR-33 sponge and these two sequences are separated by an IRES region (internal ribosome entry site) from the encephalomyocarditis virus, although the antimiR-33 sponge was not translated, the IRES region allows the translation of the reporter gene to indirectly determine the sponge expression, like a bicistronic plasmid. The expression of the EGFP reporter gene was achieved in liver cells when they were transfected with the recombinant plasmid pPEPCK-antimiR33-IRES2-EGFP and with the control plasmid pIRES2-EGFP (transfection efficiency of 20% in Hep G2 and 5% in primary mouse hepatocyte culture); however, in the control cells there was no expression of EGFP when they were transfected with the recombinant plasmid, but there was with the control plasmid (transfection efficiency of 65% in Hek293 FT) (Figure 2, Figure 3 and Figure 4).

### 3.4. antimiR-33 Governed by the PEPCK Promoter Indirectly Up-Regulate ABCA1 and ABCG1 mRNA, Only in Hepatocytes

**Figure 5:**
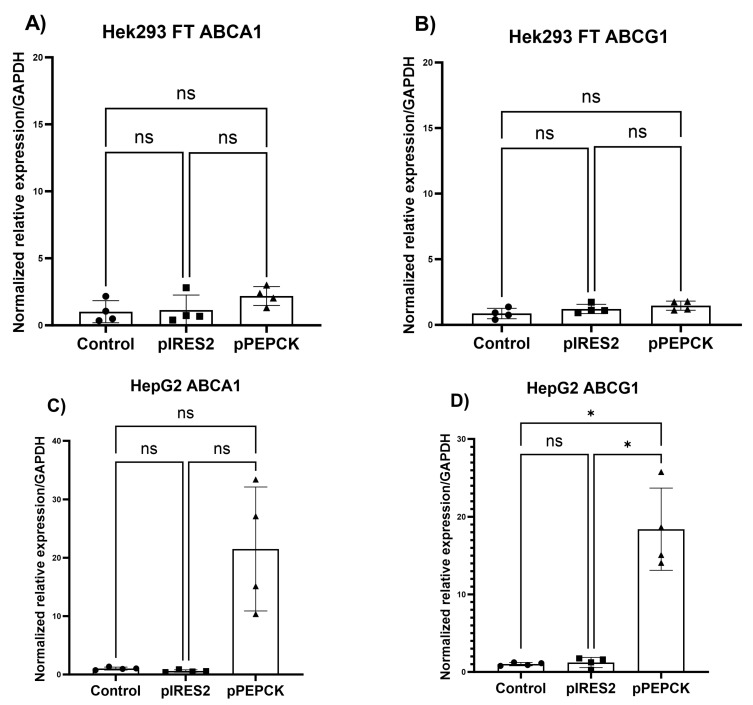
Differential expression of miR-33 target messengers: ABCA1 and ABCG1 in Hek 293 FT and Hep G2 cell lines. The cell lines were not transfected or transfected with the control plasmid pIRES2-EGFP or the recombinant plasmid pPEPCK-antimiR-33-IRES2-EGFP. Results are shown by cell line and by evaluated gene, as the mean of *n* = 4 with respective standard deviation. (**A**) ABCA1 gene in Hek293 FT, these data were analyzed by Kruskal–Wallis followed by Dunn’s post hoc tests. (**B**) ABCG1 gene in Hek293 FT. (**C**) ABCA1 gene in Hep G2. (**D**) ABCG1 gene in Hep G2. All data in B, C, and D were analyzed by Brown–Forsythe and Welch ANOVA followed by Dunnett’s post hoc tests (*p* ≤ 0.05). ns = no significantly, * = significantly difference.

Relative expression analysis of the miR-33 target messengers, ABCA1 and ABCG1, showed that the expression of ABCA1 and ABCG1 was not significantly different in the Hek293 FT cell line between the three evaluated groups (*p* = 0.173 and 0.1809, respectively) (Figure 5A,B); however, in the Hep G2 cell line, the expression of ABCA1 increased more than 21-fold, in the transfected group with the recombinant plasmid, compared with non-transfected cells and transfected with pIRES2-EGFP (Figure 5C), although this difference was not statistically significant (*p* = 0.068). In the case of ABCG1, the expression was up to 18 times higher in the transfected group with the recombinant plasmid, compared with non-transfected cells and transfected with pIRES2-EGFP (*p* = 0.0057) (Figure 5D), with a mean difference of 17.17 (SE = 2.65).

With all these results, it was found that the antimiR-33 sponge expression, through recombinant plasmid pPEPCK-antimiR-33-IRES2-EGFP, can be used to efficiently induce the expression of the miR-33 target genes, ABCA1 and ABCG1, although additional studies are required to determine the adequate reduction in miR-33, as well as to evaluate the specificity of expression in other cell types.

## 4. Discussion

CVDs continue to be the main cause of death in Mexico and worldwide, hypercholesterolemia and atherosclerosis being the main triggers [39]. miR-33 is a very important miRNA, both biologically and clinically, due to its participation in the regulation of lipid metabolism and the development of atherosclerosis [3,9,10,40]. It has been validated that the specific overexpression of miR-33 generates a decrease in the translation of ABCA1 and ABCG1 transporters, which leads to the development and/or progression of atherosclerosis [7,20,41]. Hence, in this work, the genetic construct pPEPCK-antimiR-33-IRES2-EGFP was designed and constructed to express a miRNA sponge containing two binding sites for miR-33, as a new strategy to specifically inhibit the function of this miRNA in liver.

Currently, several antagonism strategies for miR-33 have been designed as therapeutic agents. The simplest method, called anti-miRNA oligonucleotides (AMOs), and derived from ASO, uses oligonucleotides complementary to the sequence of the mature miRNA to prevent the interaction with its physiologically relevant target messengers [8]. However, the main obstacles for the transition of this technology to clinical stages are: its vulnerability to the hydrolytic action of nucleases, the fact that they cannot cross the cell membrane due to their negatively charged nature; and because they are known to trigger an immune response [42]. Another form of repression like AMOs is miRNA sponges, which also seek to block mature miRNAs, but instead of using short sequences complementary to them, DNA sequences are used, generally plasmids, which express considerable anti-miRNA transcript copy number and are less vulnerable to nuclease action [23,24]. Therefore, the use of the recombinant plasmid pPEPCK-antimiR-33-IRES2-EGFP, administered through cationic liposomes, provides a certain degree of protection to the genetic material against the hydrolytic action of the nucleases present in serum and allows a greater degree of expression of the interest gene [27].

In preclinical trials, it has been possible to suppress the effect of miR-33 using antimiR-33 ASO, thus reversing the effects of atherosclerosis in the employed models [17,19,20]. However, although these strategies have been effective in reducing miR-33 levels, given the route of administration [16,43], this remains a non-specific option, since targeting to specific cells is still deficient, in addition, there may be a low uptake efficiency and, therefore, high doses and continuous administration are required, leading to the presence of adverse effects.

Kluiver et al. [31] conducted a description of the method to construct sponge sequences for the specific inhibition of miRNAs. In this work, the miRNA sponge sequence containing two binding sites for miR-33a was designed based on this method. The use of these sponges has emerged as a potential tool to inhibit the function of miRNAs in different pathologies where they are overexpressed, or where it is sought to increase the levels of their target proteins [32,44]. Furthermore, the use of antimiRNA sponges has been related to a phenomenon that occurs naturally in mammalian cells, where a circRNA acts as an miRNA sponge and inhibits their activity [45,46].

It has been shown that, with the use of genetic constructs for gene therapy, the transcript which has more than one antisense site, but no more than six, can improve the inhibition of a given miRNA, which would produce a greater biological effect [32] and thus the number of doses required for treatment would be reduced. Therefore, with the presence of the two antisense sites in the antimiR-33 sponge sequence, miR-33 levels can be moderately but efficiently reduced, without affecting any other pathway in which they could play an important role.

The antimiR-33 sponge binding specificity and functionality for the inhibition of the miR-33 family was confirmed with the miRNAsong online software, an open-access tool for the in silico analysis of miRNA sponges, which has a regular update plan and covers 219 species, as well as more than 35 thousand mature miRNAs. There are other available web servers like STarMir software or PITA tool that can be used for sponge testing, but they present several disadvantages [32]. The results in miRNAsong showed that the designed sponge has two possible interacting ways with miR-33, in each antisense site, one was found to bind with 100% complementarity with the miR-33a; in addition, the fact that it can bind partially in a secondary way to miR-33b would ensure the inhibition of miR-33 in a more efficient way. However, it was shown that the antimiR-33 sponge can be a target for other miRNAs in both antisense sites, although, according to the ΔG values obtained (less negative), the interaction would be weak, so it is less likely that they would join with it.

To give specificity to the expression of introduced genes in association with cationic liposomes, the use of promoters from specific tissues is resorted to; in this case, the genetic construction of pPEPCK-antimiR-33-IRES2-EGFP was carried out, which contains the antimiR-33 sponge sequence as a therapeutic sequence and the EGFP as a reporter gene, governed under the transcriptional control of the PEPCK promoter, an enzyme of the gluconeogenesis pathway, so that genetic expression could be directed exclusively to the hepatocyte, without having any effect on other types of cells, as could be seen in the in vitro transfection assays. Likewise, the miRNAsong results showed that there are some miRNAs that can bind nonspecifically to the antimiR-33 sponge but most of these are generally not expressed in hepatocytes, which is where expression is directed with this genetic construct. Furthermore, as briefly mentioned above, introducing the recombinant plasmid into the target cells of an organism, in association with and protected by a liposomal vehicle, to express the antimiR-33 sponge, avoids the administration of naked RNA, which has been proven to be more vulnerable to the action of nucleases in the blood, and significantly reduces the half-life of the genetic material in circulation, so its therapeutic effect is not very effective [21].

On the other hand, a controlled inhibition of miR-33 is very important, since a decrease in miR-33a has been observed in hepatoma cells, and this inhibition is also related to a low survival rate in patients with HCC [47]. In addition, there is increasing information related to the adverse effects of antimiR-33 therapy, mainly related to obesity and insulin resistance; however, these effects were reported when the murine model used was fed with a high-fat diet, after the inhibition of this miRNA in the very long term or when miR-33 knock-out mice were used for these studies. Furthermore, several in vivo studies have also shown that the short- and long-term (but transient) inhibition of miR-33, as well as targeted inhibition in liver or macrophages, does not induce the development of the drastic obese phenotype found in miR-33 knock-out mice [21,22,48,49]. There is even a concern that the complementary or temporary chain of miR-33 (miR-33*) is the one that could be the direct cause of these deleterious effects, since it cannot be inhibited by antimiR-33 therapies [50]. In the present work, it is expected not to cause affectations, since the decrease in miR-33 would be temporary, at a moderate level, and specifically directed to hepatocytes.

As mentioned, the recombinant plasmid pPEPCK-antimiR-33-IRES2-EGFP contains the 674 bp PEPCK promoter, reported by Short et al. in 1992 [51]. Furthermore, downstream of the therapeutic agent, the EGFP gene is found; these two sequences are separated by an IRES (internal ribosome entry sequence) region of the encephalomyocarditis virus, with great importance for this plasmid design because as the antimiR-33 sponge is not translated, the IRES region allows the translation of the reporter gene (EGFP) [52], which indirectly evidenced the expression of the sequence of interest in the transfection and expression specificity assays on Hek293 FT and Hep G2 cell lines, as well as in the primary culture of mouse hepatocytes.

In the transfection assays, the specificity of expression of the antimiR-33 sponge was demonstrated only in liver cells, because the expression is regulated by the tissue-specific promoter PEPCK. For the RT-qPCR assays, CT values are used to quantify the expression of the gene of interest, using relative quantification [53]. In this work, the variation in transcription levels was determined as fold change, for which the relative quantification method was used [54]. The results of the expression of the miR-33 target messengers, ABCA1 and ABCG1, suggest that the antimiR-33 sponge is functional to indirectly increase the levels of these genes of clinical interest, as it was demonstrated by the 2^−∆∆CT^ method and this difference was statistically significant for ABCG1 (*p* = 0.0057).

Altogether, the recombinant plasmid pPEPCK-antimiR-33-IRES-EGFP could be used in other pre-clinical trials to increase the expression levels of the ABCA1 and ABCG1 transporters by blocking the miR-33 specifically in hepatocytes, since the antimiR-33 sponge can be expressed inside the cell and using its molecular machinery, so it is expected not to have undesirable side effects [55]. The intention is to implement a specific gene therapy strategy against hypercholesterolemia and atherosclerosis because there are still no fully satisfactory treatments to combat this disease that causes so many deaths worldwide; however, more research is required to determine the adequate reduction in miR-33 and the up-regulation of the transporters at a protein level, as well as to evaluate the specificity of expression in other cell types, but mainly in experimental animals. For the latter, a time-course and dose-response analysis will be considered, with the same control pIRES2-EGFP and more specific procedures to evaluate the changes in some characteristics of atherosclerosis such as inflammation, as well as cholesterol levels and changes in atherosclerotic plaque.

## 5. Conclusions

An in silico analysis of the antimiR-33 sponge sequence indicated that it may be functional to specifically repress miR-33 activity, although additional assays are required to prove it. It was shown that the therapeutic gene (antimiR-33 sponge) and the tissue-specific promoter that governs its expression (PEPCK), are correctly and fully inserted in the recombinant plasmid pPEPCK-antimiR-33-IRES2-EGFP. In addition, it was indirectly revealed that the expression of the antimiR-33 sponge is specific for liver cells and it functions to induce the expression, at the transcriptional level, of the target genes of miR-33: ABCA1 and ABCG1. This construction could be used in additional assays with an in vivo model, in order to search the first solid bases for the future development of a possible and safer gene therapy to reduce hypercholesterolemia and, therefore, atherogenic risk.

## Figures and Tables

**Table 1:** Design of the antimiR-33 sponge, with two perfect binding sites to miR-33 (Gray).

miR-33a Sequence	5′-GUGCAUUGUAGUUGCAUUGCA-3′
AntimiR-33 sponge sequence designed as duplex oligonucleotide	
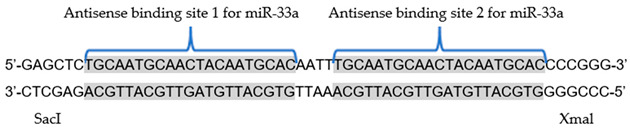	

## Data Availability

No new data were created or analyzed in this study. Data sharing is not applicable to this article.

## Supplementary Materials

The following are available online at https://www.mdpi.com/article/10.3390/cimb45090445/s1, Figure S1: Design and in silico construction of recombinant plasmid pPEPCK-antimiR-33-IRES2-EGFP, Figure S2: Characterization of recombinant plasmid pPEPCK-antimiR-33-IRES2-EGFP.
