# Supplementary material for: Targeted Expression to Liver of an antimiR-33 Sponge as a Gene Therapy Strategy against Hypercholesterolemia: In Vitro Study
Source: Curr Issues Mol Biol. 2023 Aug 24;45(9):7043–57. doi: 10.3390/cimb45090445 (PMC10527677; doi:10.3390/cimb45090445)
Supplement: Supplementary file 1 [file cimb-45-00445-s001.zip › suplementary materials.pdf]

SUPPLEMENTARY MATERIALS

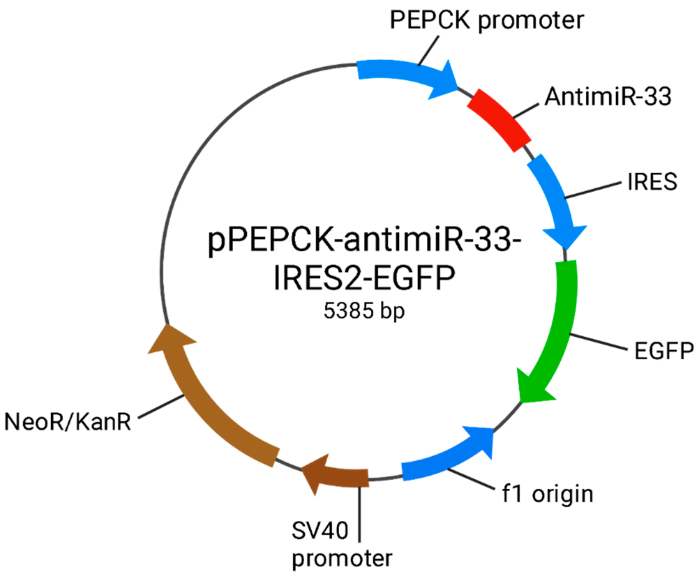

Figure S1. Design and *in silico* construction of recombinant plasmid pPEPCK-antimiR-33-IRES2-EGFP. The recombinant plasmid contains the antimiR-33 sponge and the EGFP reporter gene, under the transcriptional control of PEPCK promoter. Created with BioRender.com.

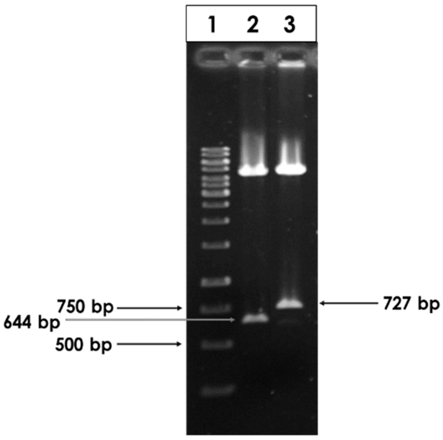

(**A**)

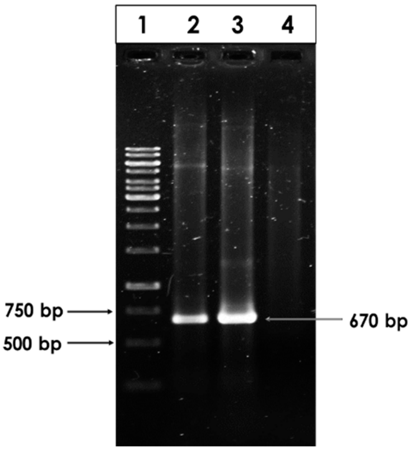

(**B**)

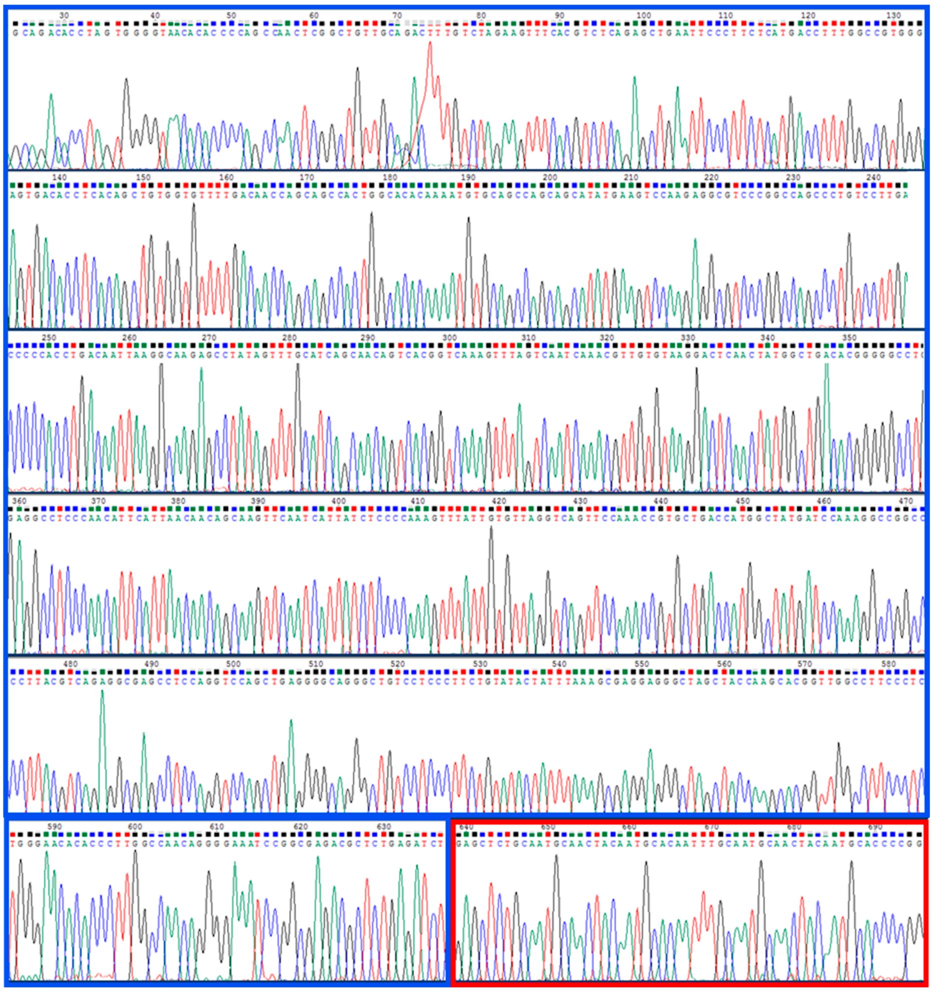

(**C**)

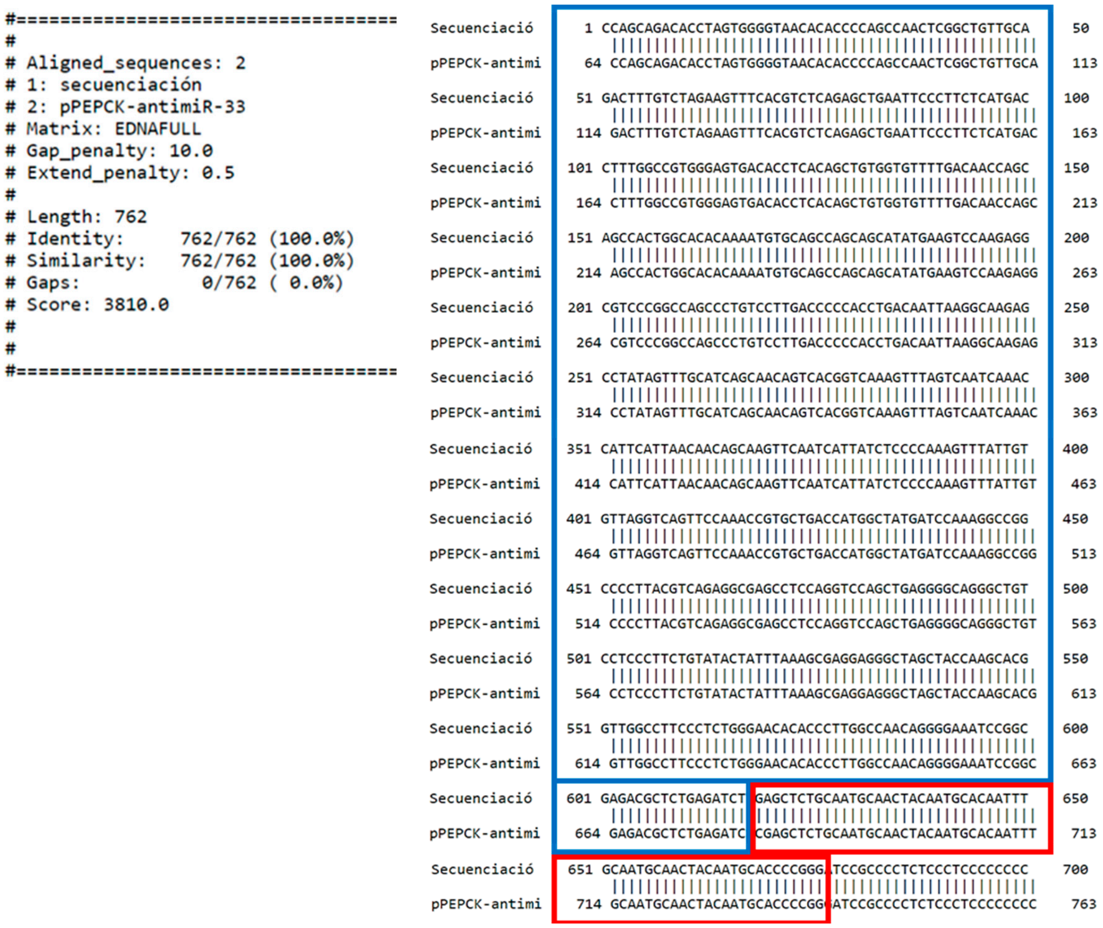

(**D**)

Figure S2. Characterization of recombinant plasmid pPEPCK-antimiR-33-IRES2-EGFP. (**A**) Electropherogram showing enzyme restriction with AseI and XmaI of the recombinant and control plasmid, 1. 1 Kb molecular size marker, 2. Parent plasmid pIRES2-EGFP (644 bp = CMV promoter and part of the MCS), and 3. Recombinant plasmid pPEPCK-antimiR33-IRES2-EGFP (727 bp = PEPCK promoter and antimiR-33 sponge); (**B**) Electropherogram showing the PEPCK promoter obtained by PCR, 1. 1 Kb molecular size marker, 2. Positive control of the PEPCK promoter, 3. Amplification of the PEPCK promoter from the recombinant plasmid pPEPCK-antimiR-33-IRES2-EGFP, and 4. Negative control of PCR reaction; (**C**) PEPCK promoter sequence and antimiR-33 sponge in the recombinant plasmid pPEPCK-antimiR-33-IRES2-EGFP, PEPCK promoter in blue and antimiR-33 sponge in red; (**D**) comparison of the designed plasmid pPEPCK-antimiR-33-IRES2-EGFP to the nucleotide sequence obtained by sequencing, PEPCK promoter (Blue square) and antimiR-33 sponge (Red square).
